# CAM in Psychiatry

**DOI:** 10.1155/2013/293248

**Published:** 2013-07-25

**Authors:** Jörg Melzer, Hans-Christian Deter, Bernhard Uehleke

**Affiliations:** ^1^Institute of Complementary Medicine and Clinic for Psychiatry and Psychotherapy, University Hospital Zurich, Raemistraße 100, 8091 Zurich, Switzerland; ^2^Clinic for Family Medicine, Naturopathy and Psychosomatics, Charité, 12200 Berlin, Germany; ^3^University of Applied Sciences Health and Sports, 10367 Berlin, Germany

## 1. Aspects of Psychiatry

In western countries, the 19th century marks a turning point for the beginning of psychiatry as an academic discipline. Since this happened and psychiatry had left the asylums at the boundaries not only of cities but medicine itself, we can find quite a row of examples concerning integration [1]. Psychiatry has integrated many treatments into the therapeutic spectrum, for example, tricyclic antidepressants, SSRI, relaxation techniques like progressive muscle-relaxation or hypnosis, cognitive-behavioural, psychoanalytic, or systemic psychotherapy, even acupuncture according to the protocol of the National Acupuncture Detoxification Association (NADA) [2, 3] or lately mindfulness-based cognitive therapy [4]. However, three aspects seem suitable to understand the openness of psychiatry for new methods: (a) the fact that since decades, professional multidisciplinarity in psychiatry is a crucial basis for the in- and outpatient treatment (e.g., psycho-, ergo-, music-, physiotherapists, nutritionists, and social workers), (b) psychiatrists today need to have a double qualification in psychiatry and psychotherapy to be able to work according to (c), Engel's biopsychosocial model, which had been adopted as a solid basis for the previously mentioned. 

## 2. Aspects of CAM

Traditional medicine has been present for centuries in different cultures around the globe (e.g., Ayurveda, Homeopathy, Kampo Medicine, Traditional European Medicine, Tibetan Medicine, and Traditional Chinese Medicine). However, with the development of modern medicine, traditional medicine was regarded as an old fashioned way of practice and theory, and consequently, traditional medicine mostly was not taught at medical schools and universities. Only few chairs or colleges for naturopathy or homeopathy in the US and some single chairs in Europe for hydrotherapy or naturopathy can be regarded as an exception to the rule in the 19th and 20th century. Nevertheless, traditional medicine remained in the cultures as a kind of folk medicine often provided by lay physicians and sometimes also by physicians with their institutions and inventions (e.g., Friedrich Bilz at Bilz'sche Naturheilanstalt, Germany, and his concept of combined naturopathy; Maximillian Bircher-Benner at Sanatorium Lebendige Kraft, Switzerland, and his Bircher-Muesli; Harvey Kellog at Battle Creek, USA, and his corn-flakes) [5, 6]. 

## 3. Research 

Interestingly, the concept of Evidence-based Medicine (EbM) [7] brought a chance for CAM and traditional medicinal systems. Maybe this derived from the fact that EbM was new for the whole medical system and activated a new orientation in research. A young generation of academic protagonists in CAM adopted the promising paradigm of EbM and hundreds of systematic reviews; meta-analysis and new randomized controlled trials (RCT) brought clinical evidence for certain CAM methods (even when the mode of action remained unclear). So, a broader scientific acceptance for certain CAM interventions was achieved. Yet, next to evidence from RCTs with high internal validity, the argument that interventions from CAM (the same accounts for psychotherapy) work by “placebo response” (or better: unspecific treatment effects) rather than specific therapeutic effects had been discussed as well [8]. The different CAM interventions, the effects of the physician/patient relationship, and other contextual factors seem to influence outcome as can be seen from various methodological settings (e.g., acupuncture versus sham acupuncture, individualized versus standardized homeopathy). It remains a challenge for research to use methods to evaluate these patient specific factors. From this point of view, collaboration and exchange between CAM with psychiatry and psychosomatic medicine seems promising.

## 4. CAM and Psychiatry

Funny enough and often overseen, there was a time for a kind of approach between CAM and psychiatry in the beginning of the 20th century. The German physician Georg Groddeck combined naturopathy with psychotherapy: he had a little naturopathic clinic in Baden-Baden and was a promising psychoanalyst in tense contact with S. Freud until they became kind of rivals over the concept of the Id [9, 10]. Another protagonist was the Swiss naturopathic physician M. Bircher-Benner who set up the system of Ordnungstherapy, which meant the combination of naturopathic somatotherapies with psychotherapy [4]. In aspects, this also had similarities with psychiatric Milieu therapy. This is no wonder, as for a time Bircher-Benner was a pupil of the Swiss psychiatrist August Forel and even adopted hypnosis from him. 

From the concept of naturopathic Ordnungstherapy derived a short and punctual contact to the new direction in psychiatry, the psychosomatic medicine (PM) in German speaking countries, which somehow criticised the domination by the biological view [11]. But in the very beginning, CAM and PM failed to find a strong academic and practical connection with each other. Yet, today one might speculate about a renewed alliance under the term mind-body medicine, in which multidisciplinarity including various forms of psychotherapy and patient-centred healthcare [12] is combined with educational aspects for the patients.

## 5. CAM in Psychiatry

CAM in psychiatry seemed as a timely topic for a special issue of the eCAM journal, since the journal had focussed in its special issues on specific CAM methods (e.g., Tai-Chi, medical mushrooms), certain diseases (e.g., obesity, diabetes mellitus), or single research questions (e.g., neurobiology of acupuncture, network pharmacology). On the other hand, patients with psychiatric disorders use CAM (mostly add-on in 20 to 50% in depression and 20 to 40% in anxiety and much less in addiction disorders [13, 14]). Therefore, it is a crucial responsibility for physicians and researchers to evaluate efficacy and safety of CAM to secure patients' safety and interests. 

Some psychiatric hospitals or departments have drawn attention for turning CAM into their daily practice. The reasons for this might be an increase in evidence for single CAM methods, the criticism of the evidence for conventional psychopharmacology [15], the strengthened awareness of its side effects [16, 17], or simply the marketing advantage in competition. However, by no mean do we expect to draw a representative picture in this issue of what is happening between psychiatry and CAM today. We present a somewhat random cross-sectional perspective among researchers in the field, deriving from the call for papers on the journals homepage and additionally contacting about 100 researchers or working groups we were aware of. 

In its mixture of papers, this issue differs from earlier publications on CAM in psychiatry, like reviews on herbal medicine [18–20] or CAM [21], guidelines incorporating, for example, St. Johns' Worth for the treatment of mild to moderate single depressive episodes based on meta-analytic evidence [22–24], or the work of the International Network of Integrative Mental Health (INIMH), founded in 2010 and reviews of its protagonists on bipolar disorders or ADHS [25, 26]. So, we hope this special issue can contribute to further discussion on CAM in psychiatry—the subject may be named integrative mental health [27], mind-body medicine [28], or integrative psychiatry [29].

For herbal medicine, the most important spectrum of EbM methods could be covered with 3 studies: N. Brondino et al. systematically review preparations from *Ginkgo* for different psychiatric disorders. In their meta-analysis, they evaluate the Gingko-treatment not only for dementia but also as add-on in schizophrenia. The systematic review of Y. W. Wong et al. on different “traditional oriental herbal medicine” in ADHS gives an idea of the difficulty tracing a cultural/national origin of some herbal preparations in Asiatic countries and the need for profound EbM research. The RCT of R. Schellenberg et al. on a *Cimicifuga* preparation in menopause cannot answer the question whether the effects are vasomotoric or psychological but shows dose-dependent efficacy for both with a slightly modified questionnaire.

Concerning acupuncture/acupressure 3 studies were included: Y.-D. Kim et al. systematically review RCTs on posttraumatic stress disorder and give a meta-analytic evaluation of 2 RCTs on acupuncture plus moxibustion and necessary recommendations for future research. The small RCT of H. Y. Ching et al. examines efficacy of standardised auricular acupressure on body weight in chronically schizophrenic, hospitalised patients—a relevant question due to metabolic side effects of neuroleptic treatment. The pilot study of P. Bosch et al. is an example of integrating individualised acupuncture add-on to psychopharmacology for sleep improvement of schizophrenic and depressed patients in daily clinical practice giving hints on moderate clinical relevance and need for further research.

Referring to relaxation techniques, 3 studies give the following picture: the systematic review of F. Wang et al. on Qigong in different conditions leads to a meta-analysis in multimorbid patients, that is, diabetes and depression and a psychosomatic reflection. The survey of M. Nedeljkovic et al. examines a side of Taiji as a medical interventions, hardly examined so far: the effect of expectations of consumers and providers. Evidence from the pilot study of R. T. H. Ho et al. on efficacy of Tai-chi on movement/functioning in chronically schizophrenic patients seems encouraging for conducting a future RCT in this clinical situation to try to improve patients' quality of life.

Light on aspects of healthcare shed 2 further studies: the observational study of E. Jeschke et al. examines care of a small network of anthroposophic physicians for depressed patients and raises questions concerning individualised versus guideline treatment. The research paper of F. W. Stahnisch et al. examines the impact of sociopolitical circumstances like the Flexner report in North America on hindering the so much needed research on CAM in the last century.

A lesson we have learned arose from a task not in focus of our integrative view: psychiatry has not yet integrated its different classification systems. We are aware of the International Classification of Diseases (ICD-10) of the World Health Organisation (WHO) or the Diagnostic Manual (DMS-IV) of the American Psychiatric Association (APA). Yet, we also had to face manuscripts based on the Chinese Classification of Mental Diseases (CCMD). Obviously, it was not only difficult but impossible for the authors to provide a solid sound comparison between CCMD and either ICD-10 or DSM-IV. Next to these classification systems common in one or the other region of the world, we would like to mention the difficulties with “traditional diagnostic systems” as well. For example, in traditional TCM, concepts of disease stand aside the previously mentioned modern classification systems. So, no publication on these two topics is presented here. We have to bear in mind that in a globalised world, much needs to be done to bridge the gap between different medical languages, classifications, and cultures in a somehow operational and confirmatory way yet, without cultural or scientific hegemony.
